# Proteome Analysis of Condensed Barley Mitotic Chromosomes

**DOI:** 10.3389/fpls.2021.723674

**Published:** 2021-08-23

**Authors:** Zdeněk Perutka, Kateřina Kaduchová, Ivo Chamrád, Jana Beinhauer, René Lenobel, Beáta Petrovská, Véronique Bergougnoux, Jan Vrána, Ales Pecinka, Jaroslav Doležel, Marek Šebela

**Affiliations:** ^1^Department of Protein Biochemistry and Proteomics, Faculty of Science, Centre of the Region Haná for Biotechnological and Agricultural Research, Palacký University Olomouc, Olomouc, Czechia; ^2^Institute of Experimental Botany of the Czech Academy of Sciences, Centre of the Region Haná for Biotechnological and Agricultural Research, Olomouc, Czechia; ^3^Department of Molecular Biology, Faculty of Science, Centre of the Region Haná for Biotechnological and Agricultural Research, Palacký University Olomouc, Olomouc, Czechia

**Keywords:** barley, chromatin, FIBRILLARIN 1, flow cytometric sorting, mass spectrometry, mitotic chromosome, perichromosomal layer, protein prediction

## Abstract

Proteins play a major role in the three-dimensional organization of nuclear genome and its function. While histones arrange DNA into a nucleosome fiber, other proteins contribute to higher-order chromatin structures in interphase nuclei, and mitotic/meiotic chromosomes. Despite the key role of proteins in maintaining genome integrity and transferring hereditary information to daughter cells and progenies, the knowledge about their function remains fragmentary. This is particularly true for the proteins of condensed chromosomes and, in particular, chromosomes of plants. Here, we purified barley mitotic metaphase chromosomes by a flow cytometric sorting and characterized their proteins. Peptides from tryptic protein digests were fractionated either on a cation exchanger or reversed-phase microgradient system before liquid chromatography coupled to tandem mass spectrometry. Chromosomal proteins comprising almost 900 identifications were classified based on a combination of software prediction, available database localization information, sequence homology, and domain representation. A biological context evaluation indicated the presence of several groups of abundant proteins including histones, topoisomerase 2, POLYMERASE 2, condensin subunits, and many proteins with chromatin-related functions. Proteins involved in processes related to DNA replication, transcription, and repair as well as nucleolar proteins were found. We have experimentally validated the presence of FIBRILLARIN 1, one of the nucleolar proteins, on metaphase chromosomes, suggesting that plant chromosomes are coated with proteins during mitosis, similar to those of human and animals. These results improve significantly the knowledge of plant chromosomal proteins and provide a basis for their functional characterization and comparative phylogenetic analyses.

## Introduction

Nuclear DNA in eukaryotes is tightly associated with various proteins to form chromatin (Fierz and Poirer, 2019). The nucleoprotein complex not only participates in DNA packaging so that it fits the small nuclear volume, but also plays an important role in functional organization of DNA in the three-dimensional nuclear space, DNA damage repair, and regulation of gene expression. It also facilitates replication and faithful transmission of hereditary information to daughter cells during mitosis, and the production of functional gametes in meiosis, which are intricate, highly dynamic and strictly controlled processes. At the beginning of mitosis and meiosis, the interphase chromatin undergoes a series of structural changes that lead to the formation of condensed chromosomes (Antonin and Neumann, 2016).

The organization of condensed chromosomes and their function is determined by a variety of proteins. Structural maintenance of chromosome (SMC) family complexes, including condensin, cohesin, and SMC5/6, modulate the chromosome structure and impact their function during mitosis (Skibbens, 2019). Replicated sister chromatids are tethered together by cohesins. In prophase, condensin II binds DNA and extrudes large initial scaffolding loops (Ganji et al., 2018). In prometaphase, after nuclear envelope breakdown, condensin I binds to chromatin and forms smaller loops for a further compaction, which are nested within the large loops produced by condensin II. Additional proteins were described as condensation factors including topoisomerase II and in mammals also chromosome-associated kinesin KIF4. Moreover, the condensation of chromosomes is facilitated by histone modifications, including phosphorylation and deacetylation (Antonin and Neumann, 2016).

Chromosome condensation was expected to be accompanied by the eviction of proteins involved in the regulation of gene expression, chromatin state, and accessibility (Martínez-Balbás et al., 1995). This was confirmed in the case of epigenetic modifiers that promote transcription (Ginno et al., 2018) and for a majority of polymerase II transcription elongation complexes (Parsons and Spencer, 1997; Ginno et al., 2018). However, repressive modifiers, some polymerase II ternary complexes, and a majority of transcription factors are retained, including core promoter-binding proteins (Parsons and Spencer, 1997; Ginno et al., 2018; Djeghloul et al., 2020). These proteins, collectively called mitotic bookmarking factors, ensure the transfer of gene regulatory information to daughter cells (Festuccia et al., 2016; Raccaud and Suter, 2018; Zaidi et al., 2018). As the accessibility of chromatin to regulatory proteins is not dramatically changed during chromosome condensation (Hsiung et al., 2015; Blythe and Wieschaus, 2016), many genes can be expressed during mitosis (Palozola et al., 2017), implying the association of various proteins and RNAs with the chromatin of condensed chromosomes.

In mammalian models, it has been shown that a perichromosomal layer covering the whole chromosome is established simultaneously with the chromosome condensation except for the centromeric region where the kinetochore complex is formed. This layer represents at least 33% of the protein mass of mitotic chromosomes (Booth et al., 2016) and consists of pre-rRNA and proteins originating mostly from nucleoli, which disassemble during prophase. Stenström et al. (2020) identified 65 nucleolar proteins at the chromosome periphery. This recruitment was temporary as some of the proteins relocated during prometaphase, and the remaining ones were recruited only after metaphase. The proteins transferred during prometaphase included the Ki-67 protein, which has been shown the main organizer of the perichromosomal layer in human and animals (Booth et al., 2014). A series of studies revealed multiple roles of the layer, which include the formation and maintenance of chromosome architecture (Takagi et al., 2016), prevention of chromosome clumping (Cuylen et al., 2016), displacement of cytoplasmic components before nuclear envelope assembly (Cuylen-Haering et al., 2020), and transport of proteins and RNAs and their distribution to daughter nuclei (Sirri et al., 2016). The key role of the perichromosomal layer in chromosome function is reflected by its highly ordered structure (Hayashi et al., 2017), which excludes the formation of this domain by a random attachment of nuclear and cytoplasmic components.

Centromeric regions are the sites for the assembly of kinetochores – large protein complexes that attach chromosomes to spindle microtubules during cell division (Cheeseman, 2014). In vertebrates, the kinetochore consists of over a 100 proteins and comprises two major interaction networks (Pesenti et al., 2018). The constitutive centromere-associated network (CCAN) has 16 subunits and remains associated with centromeric chromatin throughout the cell cycle. The Knl1, Mis12, and Ndc80 network with 10 subunit super-complexes binds to CCAN at early prophase and remains attached during the whole mitosis (Hara and Fukagawa, 2020). Interestingly, the correct function of kinetochore depends on the translocation of the NOL11, WDR43, and Cirhin complex from the nucleoli to the perichromosomal layer. This is required for the centromeric enrichment of Aurora B and the subsequent phosphorylation of histone H3 (Fujimura et al., 2020) and underlines the key role of nucleolar proteins in the function of mitotic chromosomes.

Despite the great progress achieved during the past two decades in identifying and cataloging chromosomal proteins and unraveling their function, many proteins have an unknown function and many may remain to be discovered. The pioneering studies on human cell lines reported a relatively low number of chromosomal proteins, ranging from 60 to 250 (Morrison et al., 2002; Gassmann et al., 2005; Uchiyama et al., 2005; Takata et al., 2007). The first detailed survey by Ohta et al. (2010) revealed approximately 4,000 individual proteins and introduced a bioinformatics approach for statistical analysis to prove the authenticity of protein localization. A combination of six different classifiers by machine learning turned out to be crucial because only 19% of the total identified proteins could be annotated as truly chromosomal. This approach was further developed to detect protein complexes and their relation to chromosome structure and segregation (Ohta et al., 2016a; Montaño-Gutierrez et al., 2017), mitosis-specific chromosome phosphorylation events (Ohta et al., 2016b), and components of the chromosomal scaffold (Ohta et al., 2019).

Most of the advances were made by analyzing human and animal chromosomes and very little is known about chromosomal proteins in plants. To date, proteomics studies in plants focused on interphase nuclei (Tan et al., 2007; Bigeard et al., 2014; Petrovská et al., 2014; Zeng and Jiang, 2016; Blavet et al., 2017). One of the reasons for the absence of studies on plant mitotic chromosomes may be a difficulty to obtain highly synchronized plant cell populations in mitosis. Ideally, the studies should be done on purified mitotic chromosomes as this helps to discriminate the “genuine” and functionally significant chromosomal proteins from those isolated from interphase nuclei, which escaped synchronization, and cytoplasmic proteins. However, any preparation of pure fractions of mitotic chromosomes is challenging in plants (Doležel et al., 2012; Zwyrtková et al., 2020).

Here, we report on identification of a large number of proteins from condensed plant mitotic chromosomes. Our interdisciplinary approach comprised the induction of high degree of mitotic synchrony in meristem root-tip cells, purification of chromosomes by flow cytometric sorting, in-solution DNA and protein digestion, liquid chromatography of peptides, high-resolution MS/MS, and adapted multi-classifier data analysis.

## Materials and Methods

### Chemicals

Benzonase^®^ (Cat. No. E1014), DNase I (Cat. No. AMPD1), SOLu-trypsin (Cat. No. EMS0004), dithiothreitol (DTT), iodoacetamide, and tris(2-carboxyethyl)phosphine (TCEP) were from Sigma-Aldrich (Steinheim, Germany), and NEBNext^®^ dsDNA Fragmentase^®^ was from New England Biolabs (Ipswich, MA, United States). Raffinose-modified bovine trypsin (RAF-BT) was prepared as described (Šebela et al., 2006). Chromatography solvents were of LC-MS grade. All other chemicals were from commercial sources and were of analytical purity grade if not stated otherwise.

### Flow Cytometric Chromosome Sorting for Proteomic Analysis

Suspensions of intact mitotic metaphase chromosomes were prepared as described by Lysák et al. (1999) with modifications. Briefly, root-tip meristem cells of young seedlings of barley [*Hordeum vulgare* (HORVU) L.] cv. Morex were accumulated in metaphase after treatments with 2 mM hydroxyurea for 18 h, 2.5 μM amiprophos-methyl for 2 h, and ice water (overnight). Synchronized root tips were fixed in 2% (v/v) formaldehyde at 5°C for 15 min and homogenized using a Polytron PT1300D (Kinematica AG, Littau, Switzerland) at 15,000 rpm for 13 s in LB01-P buffer (Petrovská et al., 2014). The resulting chromosome suspension was stained with 2 μg mL^−1^ 4',6-diamidino-2-phenylindole (DAPI) and analyzed at a rate of ~5,000 particles per second using a FACSAria SORP flow cytometer (Becton Dickinson, San José, United States). Sort windows were set on a dot plot of fluorescence pulse area versus fluorescence pulse width to select all seven chromosomes of barley. For proteomic analyses, samples were prepared by sorting a total of 10–11 × 10^6^ chromosomes into 15-mL Falcon tubes containing 1 mL LB01-P buffer supplemented with 5 mM phenylmethylsulfonyl fluoride. Flow-sorted chromosomes were pelleted at 2,500 rpm and 4°C for 30 min, and resuspended in ddH_2_O.

### Protein Extraction Procedure No. 1

The pellets of flow-sorted barley chromosomes were decrosslinked by incubation in 50 μL of 50 mM Tris-HCl, pH 8.0, containing 2 mM MgCl_2_, at 70°C for 9 h. This was followed by adding 50 μL of the same buffer supplemented with 8 M urea and 10 mM DTT. After adding Benzonase (250 units), DNA was digested at 25°C for 24 h. Similarly, DNase I (20 units) was applied for DNA digestion. In parallel, Fragmentase alone (20 μL) or in a combination with Benzonase (as above) was used. The digestion buffer for Fragmentase was 50 mM Tris-HCl, pH 8.0, containing 15 mM MgCl_2_, and 50 mM NaCl (pipetted in an amount of 50 μL to the chromosomal pellet). The DNA digestion with Fragmentase proceeded at 37°C for 24 h. The released proteins were recovered by precipitation with chilled acetone (1:4, v/v) at −20°C for 24 h.

### Gel Electrophoresis

Protein precipitate from the extraction step (procedure no. 1) was dissolved in 25 μL of Laemmli sample buffer and kept at 60°C for 30 min. Sodium dodecylsulfate polyacrylamide gel electrophoresis (SDS-PAGE) was performed with 10% T/3.3% C resolving and 4% T/3.3% C stacking 1-mM thick vertical gels following a standard protocol (Laemmli, 1970) and using a Mini-Protean II apparatus (Bio-Rad, Hercules, CA, United States). %T stands for the total monomer concentration (in g per 100 mL) and %C stands for weight percentage of crosslinker (*N*,*N*ʾ-methylenebisacrylamide). The whole protein sample (25 μL) was applied to a sample well at the top of the stacking gel. Electrophoresis was run at 110 V until the marker dye reached the bottom of the resolving gel. Gel staining employed a standard protocol with 0.025% w/v Coomassie Brilliant Blue G-250 in 40% v/v methanol–10% v/v acetic acid (background destaining by 5% v/v methanol–7% v/v acetic acid). Gel images were obtained using an ImageScanner device and Lab Scan 5.0 software (Amersham Biosciences, Uppsala, Sweden).

### In-Gel Digestion of Proteins

The sample lane was cut horizontally into 17 sections representing protein fractions (12 stained bands and 5 less stained larger areas) of a different molecular mass. After destaining using 50 mM NH_4_HCO_3_ in 50% v/v acetonitrile (ACN) for 45 min, proteins were in-gel reduced by 10 mM DTT in 100 mM NH_4_HCO_3_ and then alkylated by 55 mM iodoacetamide in 100 mM NH_4_HCO_3_ (Shevchenko et al., 2006). In-gel digestion was performed using RAF-BT (Šebela et al., 2006). Peptides were extracted from the digests with 5% v/v formic acid (FoA)/ACN, 1:2, v/v (Shevchenko et al., 2006), recovered in test tubes after solvent evaporation in a vacuum centrifuge, and finally purified using C18-StageTips (Rappsilber et al., 2007).

### In-Solution Digestion of Proteins No. 1

The entire precipitate from extraction procedure no. 1 was dissolved in 40 μL of 100 mM triethylammonium bicarbonate, pH 8.0, containing 6 M urea and 2 M thiourea. The protein content was then assayed by the bicinchoninic acid method (Smith et al., 1985) after a sample aliquot dilution to decrease the urea concentration to 3 M. Proteins were reduced by TCEP (5 mM, 23°C, 45 min) and alkylated using iodoacetamide (50 mM, 23°C, 30 min). In-solution digestion with RAF-BT was subsequently done using a protein-to-trypsin molar ratio of 20:1.

In-solution digests were fractionated using the StageTips (Rappsilber et al., 2007) containing Empore^™^ Cation Exchange-SR extraction disks 2251 (3 M Bioanalytical Technologies, St. Paul, MN, United States) or by reversed-phase chromatography in a microgradient (MG) device (Franc et al., 2013a,b). The cation-exchange separation was performed using a stepwise concentration gradient of ammonium acetate (25 mM, 50 mM, 75 mM, 125 mM, and 200 mM) when the total elution was achieved by 5% v/v NH_4_OH in 80% v/v ACN. The separate peptide fractions were then recovered in test tubes after solvent evaporation in a vacuum centrifuge and purified using the StageTips with Empore™ C18 extraction disks 2215 (3 M Technologies).

### Protein Extraction and In-Solution Digestion Procedure No. 2

A suspension containing 10 × 10^6^ flow-sorted barley chromosomes was repeatedly mixed with 150 μL of mass spectrometry (MS)-quality water for washings. The solid material was collected by a brief centrifugation. Next, the pellet was suspended in 40 μL of 50 mM Tris-HCl, pH 8.0, containing 2 mM MgCl_2_ and kept at 70°C and 850 rpm for 5 h. Proteins were denatured by the addition of 20 μL of the same buffer containing 8 M urea and 10 mM DTT. The mixture was incubated at 23°C for 1 h before adding 1 μL (250 units) of Benzonase and kept at 23°C without shaking for 18 h. Disulfide reduction was achieved by the addition of 15 μL of 5 mM TCEP and incubation at 23°C for 45 min. This was followed by alkylation of cysteine thiols by adding 15 μL of 50 mM iodoacetamide in 50 mM Tris-HCl, pH 8.0, and incubating at 23°C for 30 min. Protein digestion was performed using 1 μg of SOLu-trypsin in an overall volume of 240 μL of the 50 mM Tris-HCl buffer, pH 8.0, containing MgCl_2_ at 37°C and 350 rpm for 18 h. The digestion was stopped by adding 2 μL of 50% v/v FoA.

The second sample was the original root-tip homogenate containing chromosomes as used for chromosome flow sorting, and the third sample was a chromosome-depleted fraction (i.e., a homogenate from which chromosomes were removed by flow cytometric sorting). Cell lyzate proteins were obtained from 1 mL of the extract in a 5-mL tube using acetone precipitation (1:4, v/v) at −20°C for 24 h and centrifugation at 20,000 g and 4°C for 15 min. The pellet was then suspended in 1 mL of fresh acetone, transferred into a 1.5-mL tube, and collected by centrifugation as above. Further processing of the additional samples followed the protocol for chromosomes with the initial washing step omitted in case of the original root-tip homogenate.

### Peptide Quantification Assay

The acidified peptide mixture from procedure no. 2 was spun down at 10,000 g for 15 min and the supernatant was transferred into a new tube. Then, the tryptophan content in the peptides was determined using a microarray fluorescence reader Synergy MX (BioTek Instruments, United States) as published by Wisniewski and Gaugaz (2015). Samples of 200 μL were loaded into microtitration plate wells. The instrument parameters were as follows: excitation wavelength of 295 nm and bandwidth of 9.0 nm; emission wavelength of 350 nm and bandwidth of 20.0 nm; gain of 75 units, 10 reads; 20°C; and integration time of 50 μs. The calibration solutions contained 0.01–5.0 μg μL^−1^ tryptophan in the sample buffer with urea. Peptide amounts in the assayed samples were calculated using the assumption that HORVU proteins contain on average 1.95% tryptophan by mass (derived from the UniProt barley protein database, see below for details).

### Microgradient Separation of Peptides

Tryptic peptides from the digests were first chromatographed using a MG device (Franc et al., 2013a,b). The peptides in an amount of 4 μg were loaded into an equilibrated microcolumn (250 μm i.d. × 30 mM) made of Kinetex EVO C18 2.6 μm core-shell particles (Phenomenex, 00G-4,725-E0) and desalted by washing with 25 μL of 0.1% v/v TFA. Then, the retained peptides were eluted by a stepwise gradient of 8, 12, 16, 20, 24, 28, 36, and 48% v/v ACN in 20 mM NH_4_HCO_3_ aspirated into the gas-tight syringe. The eluate was collected in seven consecutive 4-μL fractions. Each fraction was then diluted by 21 μL of 5% v/v FoA for the subsequent MS analysis.

### Mass Spectrometry of Peptides

Nanoflow liquid chromatography-tandem mass spectrometry (nLC-MS/MS) analyses were performed on a maXis UHR-Q-TOF mass spectrometer equipped with a nanoelectrospray ion source (Bruker Daltonik) and connected to a Dionex UltiMate3000 RSLCnano liquid chromatograph (Thermo Fisher Scientific, Germering, Germany). Each sample was measured in two runs and the data were pooled. The experimental setup including the reversed-phase analytical column, pre-column, composition of mobile phases, flow rates, gradient programming, and other automated MS and MS/MS data acquisition parameters was the same as described previously (Chamrád et al., 2014).

### Data Analysis and Annotation

Raw data were converted into Mascot generic format-formatted files and processed for database searches using PEAKS Studio 10 (Bioinformatics Solutions, Waterloo, ON, Canada). The search parameters were as follows: mass tolerance for precursor ions and fragments – 50 ppm and 0.05 Da, respectively; enzyme – trypsin (semispecific); the number of missed cleavages – 2; allowed modifications per peptide – up to 3; variable peptide modifications – Met oxidation, Asn/Gln deamidation, protein N-terminal acetylation; and fixed peptide modification – Cys carbamidomethylation. The sequence databases used were barley (HORVU) proteome database downloaded from the UniProtKB (https://www.uniprot.org, 11/10/2020, Proteome ID UP000011116, 189,799 entries; International Barley Genome Sequencing Consortium et al., 2012) and cRAP contaminant database (downloaded from https://www.thegpm.org/crap/ on 11/10/2020). The false discovery rate was set at 1% as a positivity threshold for the peptide-spectrum match plus peptide and protein sequence matches. At least one unique peptide was required for positive protein identification and only the first identification (ID) with the highest –logP score for each protein group was used for the subsequent data evaluation.

The obtained list of IDs matching the set of barley protein sequences was then searched against the UniProtKB/Swiss-Prot database to find *Arabidopsis thaliana* (ARATH) homologs by blastp (protein-protein BLAST; Altschul et al., 2005). Then, the available information on the cellular localization, related gene ontology (GO) terms, molecular mass, and sequence length for each Arabidopsis protein accession was acquired *via* UniProtKB Retrieve/ID mapping tool. A limit of 70% sequence homology was set up for the further search on UniProtKB protein localization information for Arabidopsis homologs. The whole protein FASTA-formatted file was reduced into partial files of 400 IDs for the application of other bioinformatics tools, such as NucPred (Brameier et al., 2007), Localizer (Sperschneider et al., 2017), CELLO2GO (Yu et al., 2014), and WegoLoc (Chi and Nam, 2012). In Localizer, the input was specified as “full plant sequences.” The plant BaCelLo dataset and default settings were used in WegoLoc. In CELLO2GO search parameters, the eukaryotic organism option was selected. Also, matching GO terms and other information were obtained by searches using DAVID Functional Annotation Tool (Huang et al., 2009).

### Evaluation of Nuclear or Chromosomal Localization

All data obtained from the databases and bioinformatics tools were merged using Perseus v.1.6.10.45 (Tyanova et al., 2016) and further processed in Microsoft Excel 2016. Six groups reflecting the prediction results and UniProtKB information were established to categorize the identified proteins (Search S1). Protein IDs yielding information on a nuclear/chromosomal localization in more than two prediction tools, which possessed a positive record on their nuclear origin in UniProtKB, were marked as “NUCLEAR.” Those IDs with more than two nuclear prediction hits and lacking any UniProtKB information on nuclear localization were grouped as “PREDICTED NUCLEAR.” Proteins labeled as nuclear/chromosomal by two prediction tools with a reliable record in UniProtKB were classified as “POSSIBLY NUCLEAR.” The group “DISCREPANCY UNIPROT” contained IDs with non-nuclear UniProtKB localization information and more than two positive nuclear/chromosomal localization hits from the prediction tools. The group “DISCREPANCY PREDICTION” refers to protein IDs labeled as nuclear in UniProtKB and yielding less than two positive hits from the prediction tools. Finally, proteins classified in the “CYTOSOLIC” group were assigned according to information available on their subcellular localization in UniProtKB for HORVU or the corresponding ARATH protein accessions by searching with tags “cytos,” “cytop,” “mitoch,” “memb,” and “recept.” One positive hit for nuclear localization was a maximum for this group. The following criteria were used to filter out positive nuclear/chromosomal localizations: Localizer – predicted nuclear localization; NucPred – prediction score ≥ 0.50; WegoLoc – predicted localization contains the tag “nucl”; and CELLO2GO – the predicted localization (CP) result contains the tag “nucl” or “chromo.” The UniProtKB HORVU IDs and their ARATH homologs were searched for the tags “chromos,” “chromat,” and” “nucl” in the “Subcellular location (CC)” information provided in the database entry. Information on protein domains was obtained using CD-Search (Marchler-Bauer and Bryant, 2004; default settings) and barley FASTA sequences.

Each protein containing at least one functional domain was scored using an in-house made database of domains (inspired by Ohta et al., 2010) based on experiments following the in-solution digestion procedure 2 and MG peptide separation. Finally, it contained 869 domains. Those domains bound to the protein ID groups “NUCLEAR,” “PREDICTED NUCLEAR,” and “POSSIBLY NUCLEAR” were attributed as nuclear. Domains related to “CYTOSOLIC” proteins were considered false. Each domain for a protein ID was then scored for these attributes. Domains not included in the database were marked as unknown. Comprehensive data combining nuclear prediction hits, information on protein localization in the UniProtKB, and the domain score were re-evaluated (Search S2). Protein IDs with more than three nuclear prediction hits plus the existing nuclear localization information in UniProtKB (barley accessions) and true domain attribute were “NUCLEAR.” The same score but the existing nuclear localization information in UniProtKB for ARATH homolog only resulted in “NUCLEAR (BLAST)” classification. Proteins lacking any domain information were classified in the group “UNSUFFICIENT CD INFO.” Those with less than three nuclear prediction hits were denoted as “POSSIBLY NUCLEAR.” Missing or non-nuclear localizations found for barley and ARATH accessions in the corresponding UniProtKB/Swiss-Prot entries were evaluated as “DISCREPANCY UNIPROT.”

### Generating Barley EYFP-FIB1 Reporter Line

The CDS sequence of barley *FIBRILLARIN 1* (*FIB1*; HORVU6Hr1G091860), cultivar Golden Promise, was amplified to generate the *ZmUBI1::EYFP-FIBRILLARIN1::T35S* fusion construct. The amplification was achieved with cDNA obtained by a reverse transcription (Transcriptor High Fidelity cDNA Synthesis Kit; Roche) using total RNA isolated from roots (RNeasy kit; Qiagen) with the following primer pair: 5'-ATGAGGGCTCCCATGAGAGG-3' and 5'-CTTTTGCTTCTTGGGCATCCTGT-3', including the stop codon. *FIB1* CDS was then reamplified with another primer pair 5'- GGGGACAACTTTGTATAATAAAGTTGTTCACTTTTGCTTCTTGGGCATCC-3' and 5'- GGGGACAGCTTTCTTGTACAAAGTGGTAATGAGGGCTCCCATGAGAGG-3' containing the attB sites and cloned *via* BP reaction into a *pDONR-P2r-P3* vector by Gateway cloning strategy (Gateway^™^). The final expression cassette, including *ZmUBI1* promoter, *EYFP-FIB1*, and *T35S* terminator, was subcloned by multisite LR reaction combining three entry vectors *pEN-L4-UBIL-R1*, *pEN-L1-Y-L2*, and *pDONR-P2r-P3* with *FIB1* CDS into the *pH7m34GW* destination vector. All constructs assemblies were verified by Sanger sequencing.

The full construct in *pH7m34GW* vector was transformed into *Agrobacterium tumefaciens* strain AGL1. For barley transformation, immature embryos of the cultivar Golden Promise were dissected and transformed according to the previously described protocol (Marthe et al., 2015). Regenerated plants were genotyped for the presence of *hptII* gene, conferring resistance to hygromycin, by PCR with primer pair 5'-GACGTCTGTCGAGAAGTTTCTG-3' and 5'-CGAGTACTTCTACACAGCCATC-3'. The presence of EYFP-FIB1 fusion protein *in planta* was confirmed by the confocal microscopy using a Leica TCS SP8 STED3X microscope (Leica Microsystems, Wetzlar, Germany), equipped with an HC PL APO CS2 20 ×/0.75 DRY objective, hybrid detectors (HyD), and the Leica Application Suite X (LAS-X) software version 3.5.5 with the Leica Lightning module (Leica, Buffalo Grove, IL, United States).

### Isolation of Mitotic Chromosomes for Microscopic Analyses

Preparation of suspensions of mitotic metaphase chromosomes and flow cytometric chromosome sorting was done as described above for the proteomic analyses. However, chromosome suspensions were prepared in LB01 buffer (Doležel et al., 1989) from barley cv. Golden Promise and EYFP-FIB1 transgenic plants, and 10^5^ chromosomes were flow sorted into 25 μL of LB01 buffer. 10 μL of the flow-sorted chromosome suspension was pipetted into a 10-μL drop of P5 buffer (Kubaláková et al., 1997) on poly-lysine coated microscopic slides (Thermo Scientific^™^), air dried for up to 15 min, and stored at −20°C until use. To evaluate the effect of RNA removal, RNase A (Sigma Aldrich) was added to 100 μL aliquots of the flow-sorted chromosome suspensions in LB01 to a final concentration of 0.01 ng μL^−1^ and incubated for 30 min at 16°C prior to pipetting into microscopic slides.

### Isolation of Interphase Nuclei for Microscopic Analyses

For the isolation of root-tip meristem cell nuclei, both Golden Promise and EYFP-FIB1 transgenic seeds were surface sterilized as described (Marthe et al., 2015), cold stratified for 2 days at 4°C on a wet paper towel, and germinated for 2 days at 24°C in dark. Suspensions of cell nuclei were prepared following a previous protocol (Doležel et al., 1992) with modifications. Briefly, roots of the young seedlings were fixed in 3% (v/v) formaldehyde in 10 mM Tris buffer with additives (pH 7.5; Doležel et al., 2011) for 15 min on ice plus 5 min on ice/vacuum (500 mBa). Then, they were washed twice in the same buffer for 10 min on ice. About 30 root tips were cut with a razor blade and homogenized in 500 μL P5 buffer (Kubaláková et al., 1997) using Polytron PT1300D homogenizer (Kinematica AG) at 15,000 rpm for 13 s. The homogenate was filtered through a 30 μm nylon mesh and centrifuged at 2,000 g and 4°C for 10 min. The supernatant was removed and the pellet containing nuclei was resuspended in 100 μL of the P5 buffer. About 10 μL of the suspension was pipetted into poly-lysine coated slides (Thermo Scientific^™^), air dried for up to 15 min, and stored at −20°C.

### Immunostaining and Microscopy

The immunostaining was performed as described (Jasenčáková et al., 2001). EYFP-FIB1 was detected with primary mouse antisera against FIB1 (1:100; ab4566; Abcam) and secondary antibodies goat anti-mouse-Cy5 (Alexa Fluor^®^ 647; 1:300; A21235; Invitrogen) or with a goat anti-mouse-Cy3 (Alexa Fluor^®^ 546; 1:300; A-11003; Invitrogen) for nuclei or metaphase chromosomes, respectively. Alternatively, EYFP-FIB1 on metaphase chromosomes was detected with rabbit antisera against GFP (1,100; ab290; Abcam) recognizing also EYFP and secondary antibodies goat anti-rabbit-Cy3 (Alexa Fluor^®^ 647; 1:300; A-11010; Invitrogen) for metaphase chromosomes. Nuclei and chromosomes were counterstained with DAPI dihydrochloride (1 μg mL^−1^) in a Vectashield medium (Vector Laboratories).

Microscopic images were acquired using a Leica TCS SP8 STED3X confocal microscope (Leica Microsystems, Wetzlar, Germany), equipped with an HC PL APO CS2 63 ×/1.40 Oil objective, hybrid detectors, and the LAS-X software version 3.5.5 with the Leica Lightning module (Leica, Buffalo Grove, IL, United States). Confocal images were captured separately in sequential scans, to avoid spectral mixing, using 405 nm (DAPI), 508 nm (EYFP), 557 nm (Alexa Fluor^®^ 546), and 594 nm (Alexa Fluor^®^ 647) laser lines for excitation and appropriate emission spectrum. Pictures were processed in Adobe Photoshop version 12.0 (Adobe Systems).

## Results

### Gel-Based Identification of Barley Chromosomal Proteins

**Figure 1:**
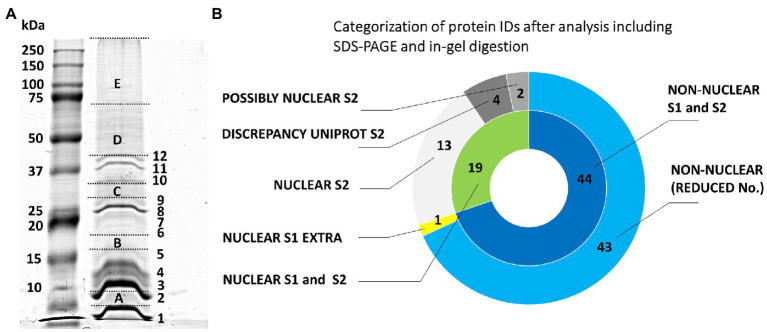
Evaluation of the origin of proteins from mitotic chromosomes identified by GeLC-ESI-Q-TOF MS/MS. **(A)** SDS-PAGE of extracted barley chromosomal proteins (Coomassie Brilliant Blue staining). The separation was achieved in a 10% T/3.3% C resolving polyacrylamide gel. Left lane, protein marker 10–250 kDa (Kaleidoscope Standards, Bio-Rad); right lane, chromosomal proteins. The excised gel fractions and bands are labeled by capital letters A-E and numbers 1–12, respectively; this labeling is used in Supplementary Table S1 for reference. **(B)** The nested pie chart shows information on the possible nuclear or non-nuclear localization of all identified proteins and their distribution into categories reflecting results of a two-round search approach (S1 and S2) utilizing predictors based on data from gene ontology prediction tools, UniProtKB database annotations and conserved domain searches (NCBI CDD database). The principle of S1 and S2 sorting is elucidated in Materials and Methods. The inner ring shows combined results of the two data search and evaluation procedures. The area labeled “NUCLEAR S1 and S2” refers to the consistently obtained attributes NUCLEAR, PREDICTED NUCLEAR/DISCREPANCY UNIPROT, and POSSIBLY NUCLEAR. The outer ring shows a protein distribution based on the procedure S2 plus an additional non-overlapping hit obtained using S1 (“NUCLEAR S1 EXTRA”). The label “NON-NUCLEAR (REDUCED No.)” refers to subtraction of the non-overlapping hit from the total number of non-nuclear identification.

Our initial experiments followed the protocol used by Petrovská et al. (2014) and Chamrád et al. (2018) to characterize the proteome of barley interphase nuclei. Their procedure included a heat-treatment, nuclease-assisted protein extraction, SDS-PAGE, in-gel proteolytic digestion, and MS/MS-based protein identification. The protein extraction step was facilitated by heat-induced disruption of formaldehyde cross-links to dissociate nuclear/chromosomal proteins from their complexes with DNA. The protocol yielded only 63 barley protein IDs (Supplementary Table S1) using 11 million chromosomes. Even though this number was much lower than expected, the electrophoretic pattern (Figure 1) was typical for chromosomal/nuclear preparations with distinct histone bands (Ohta et al., 2010; Petrovská et al., 2014). A majority of the identified proteins had a nuclear/chromosomal localization and related functions. This group included histones and also ribosomal proteins (assigned mostly as non-classified as well as cytosolic proteins according to their localization) and a few DNA/RNA-binding proteins. Other protein IDs included, e.g., abundant enzymes representing components of energy metabolism pathways (glycolysis and oxidative phosphorylation).

### Gel-Free Approaches Including Fractionations of Peptide Mixtures

We suspected that the low yield of protein IDs was related to a low protein input (10 million barley chromosomes provided an average protein mass of 4.4 μg). Therefore, the gel-based procedure was replaced by a gel-free protocol. Moreover, DNA digestion was performed differently using a set of nucleases comprising DNase I, Benzonase, and Fragmentase, the latter two were also combined in a single reaction mixture. The recovered proteins were then subjected to tryptic proteolysis and the resulting peptides were fractioned on a strong cation exchanger prior to nanoflow liquid chromatography (nLC)-electrospray ionization (ESI)-MS/MS. Table 1 shows an overview of all experiments, which are documented in Supplementary Table S2. The best results with regard to the number of protein IDs in a single experiment were obtained with the protocol using Benzonase (1169–1531 proteins). This enzyme was employed in all subsequent experiments.

**Table 1:** A summary of the results of nLC-ESI-MS/MS analyses yielding protein identification after previous peptide fractionation using strong cation exchanger.

Sample processing prior to before SCX	Matched peptide MS/MS spectra	Mean sequence coverage	Matched peptides	Unique matched peptides	Protein IDs	Possibly nuclear proteins^a^		Nuclear proteins (BLAST)^a^		Nuclear proteins^a^	
						#	%	#	%	#	%
Benzonase and in-sol digestion	60360	20.2	7491	4810	1169	17	1.5	17	1.5	47	4.0
Fragmentase and in-sol digestion	23705	17.8	4354	2943	815	5	0.6	13	1.6	25	3.1
Fragmentase+Benzonase and in-sol digestion	13232	17.1	1981	1,346	435	6	1.4	9	2.1	22	5.1
DNase I and in-sol digestion	67819	18.9	6764	4210	1090	15	1.4	17	1.6	57	5.2
Benzonase and in-sol digestion	37137	15.2	6047	4243	1216	11	0.9	22	1.8	35	2.9
Benzonase and in-sol digestion^b^	50659	19.8	7577	5029	1209	10	0.8	19	1.6	47	3.9
Benzonase and in-sol digestion (no precipitation)	24609	13.2	6873	4885	1436	13	0.9	18	1.3	46	3.2
Benzonase and in-sol digestion (no precipitation)^b^	58019	15	7186	5387	1531	13	0.8	18	1.2	42	2.7
Overall	135009	14.2	18795	13237	4139	31	0.7	62	1.5	143	3.5

a Nuclear localization in data search S2 as attributed to protein IDs using predictors (see Materials and Methods and Supplementary Table S2).

b Extraction of chromosomes was performed at pH 9 otherwise it was pH 7.

**Figure 2:**
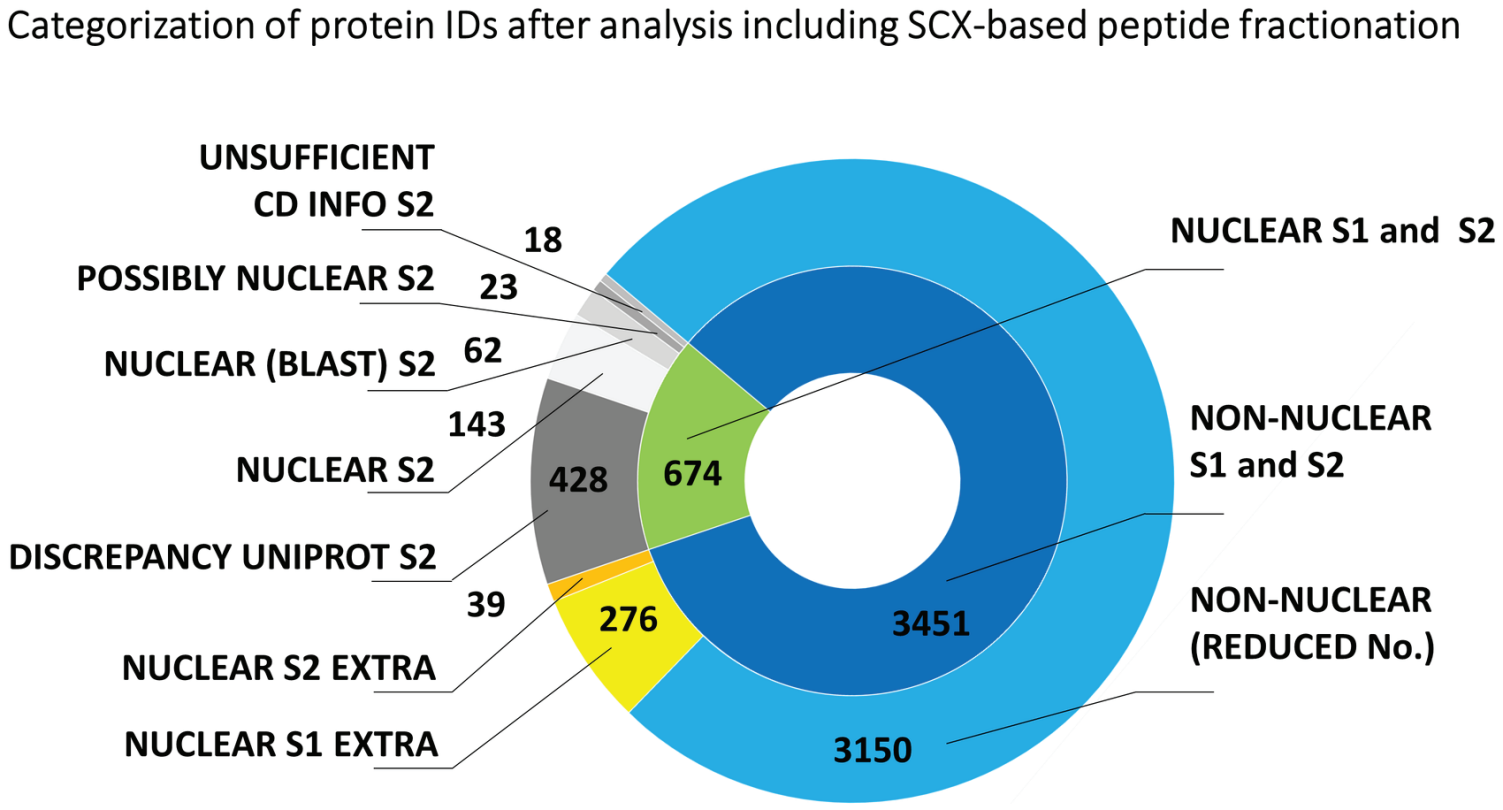
Evaluation of the origin of proteins from mitotic chromosomes identified by nLC-ESI-Q-TOF MS/MS with SCX fractionation of tryptic peptides. The nested pie chart shows information on the possible nuclear or non-nuclear localization of all identified proteins and their distribution into categories reflecting results of a two-round search approach (S1 and S2). The principle of S1 and S2 sorting is provided in Materials and Methods. See the legend to Figure 1 for elucidation of the attributed categories.

Figure 2 shows the predicted nuclear or non-nuclear localization of all identified proteins attributed in the two-round search approach referred to as S1 and S2 here. Database searches provided an overall number of 4139 protein IDs by combining individual datasets (Supplementary Table S3). A total of 674 proteins might be considered nuclear/chromosomal utilizing predictors based on data from gene ontology prediction tools, UniProtKB database annotations, and conserved domain searches. The more stringent search approach S2, which additionally considered information on the presence of a verified nuclear domain in the sequence of each identified protein, clearly confirmed 228 nuclear/chromosomal hits (143 + 62 + 23) and additional 485 entries (428 + 18 + 39) were found less plausible for classification in this category. Some of the latter IDs could not be verified by nuclear domain in S2 search (18 items) or consistent results in both S1 and S2 search (39 items). The reason resides, namely, in a discrepancy found for their localization in the UniProtKB database (i.e., they are not denoted as nuclear – 428 items).

The Panther GO (gene ontology) classification tool was applied to evaluate the identified 674 nuclear/chromosomal barley proteins (including those with the localization annotation discrepancy in UniProtKB) as regards to the attributed protein class name. Arabidopsis homologs (636 in total) were reduced to 293 unique Arabidopsis database entries for the GO classification search referring to 405 original barley proteins IDs (Supplementary Table S3). Almost two-thirds of the evaluated IDs belonged to nucleic acids-binding proteins including histones, replication factors, and various DNA/RNA processing enzymes, such as helicases, ligases, methyltransferases, topoisomerases, chromatin-remodeling complex ATPase, DNA-directed RNA polymerase subunits, and others. SMC proteins (including cohesins and condensins) were represented by 13 items. Approximately 15% of the IDs were ribosomal proteins, ribosome biogenesis regulators, and translation factors. Chromatin proteins and gene-specific transcription regulators represented roughly 5%. Other attributed nuclear/chromosomal proteins were, e.g., kinetochore proteins, nucleosome assembly proteins, importin, ubiquitin, and ubiquitin-related enzymes.

**Figure 3:**
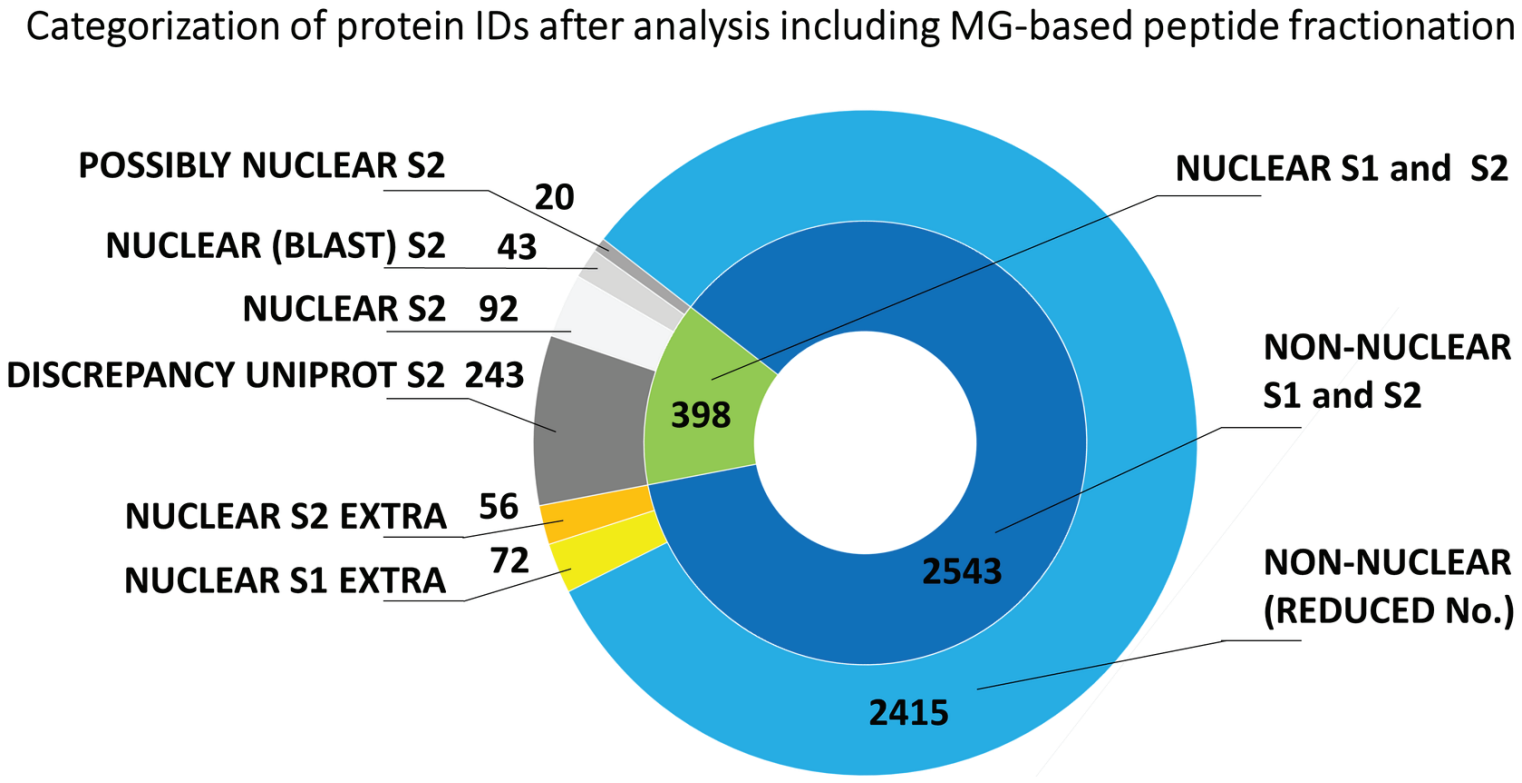
Evaluation of the origin of proteins from mitotic chromosomes identified by nLC-ESI-Q-TOF MS/MS with MG fractionation of tryptic peptides. The nested pie chart shows information on the possible nuclear or non-nuclear localization of all identified proteins and their distribution into categories reflecting results of a two-round search approach (S1 and S2). The principle of S1 and S2 sorting is provided in Materials and Methods. See the legend to Figure 1 for elucidation of the attributed categories.

Another set of experiments involved peptide fractionation using a C18 reversed-phase MG device (Moravcová et al., 2009). This approach has repeatedly been shown very helpful and efficient for a pre-separation of peptides from digests prior to nanoLC-matrix-assisted laser desorption/ionization-MS/MS or nanoLC-ESI-MS/MS analysis (Franc et al., 2013a,b). In that case, each analyzed peptide sample was first separated into seven fractions that were individually subjected to nanoLC-ESI-MS/MS. The obtained results are summarized in Figure 3. The total number of unique barley protein IDs was 2941 (Supplementary Table S4), from which 398 might be considered nuclear/chromosomal based on the bioinformatics data processing S1 + S2 as already mentioned above using UniProtKB database and prediction tools referring to the appropriate conserved protein domains and attributed gene ontology terms. The search approach S2 confirmed 155 nuclear/chromosomal hits (92 + 43 + 20). Additional 299 entries (243 + 56) were found less plausible for classification in this category, from which the number 56 were inconsistently retrieved results in both S1 and S2 search. A repeated application of the MG separation showed 1193 reproducible protein IDs. They were present in at least two biological replicates, see below, from which 144 were classified as nuclear/chromosomal.

The consensual number of 398 barley protein IDs provided 371 Arabidopsis homologs, which were reduced to 263 unique Arabidopsis database entries for the GO classification search referring to 252 original barley protein IDs (Supplementary Table S4). Again, a majority of the evaluated IDs (54%) belonged to nucleic acids-binding proteins including histones, replication/transcription/splicing factors, and various DNA/RNA processing enzymes, such as helicases, ligases, methyltransferases, topoisomerases, chromatin-remodeling complex ATPase, DNA-directed RNA polymerase subunits, and others. SMC proteins were represented by six items. About 16% were ribosomal proteins and translation factors. Chromatin proteins and gene-specific transcription regulators represented roughly 4%. Other attributed nuclear/chromosomal proteins included nucleosome assembly proteins, a kinetochore protein, transporters, and ubiquitin-related enzymes.

### Enrichment of Nuclear/Chromosomal Proteins

**Figure 4:**
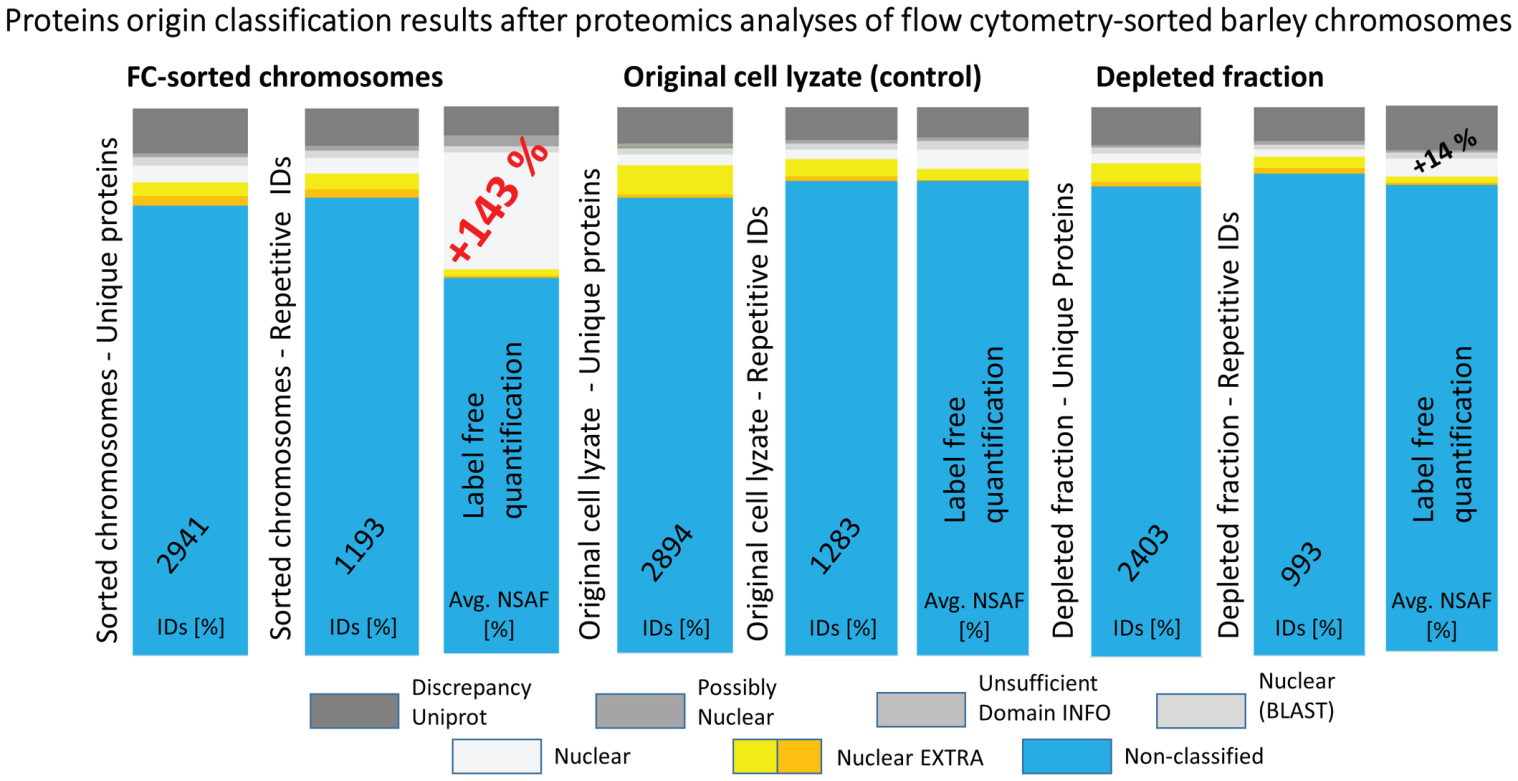
A summary of results obtained from repeated experiments with different starting biological materials. The bar plots show a comparison of protein identification results obtained using MG separation of peptides from tryptic digests followed by nLC-ESI-Q-TOF MS/MS. Three types of biological material were used for the proteomics analyses: flow cytometry-sorted barley chromosomes, original plant cell lyzates (as a control), and depleted fractions after the flow cytometry. Repetitive IDs refer to repeated experiments, where the counted hits were obtained for at least two biological replicates (three biological replicates were analyzed in total, each was run in two technical replicates). The graphics depict percentages of the protein ID categories attributed in S2 search and the corresponding NSAF values.

The experimental workflow with MG pre-separation of peptides was applied to three different sample types: (1) flow-sorted barley chromosomes, (2) original root-tip homogenate as a control, and (3) chromosome-depleted homogenate (chromosomes were removed by flow cytometric sorting). Every sample type was analyzed in three biological replicates and each of them in two technical replicates. The results are summarized in Figure 4. Our analyses considered only proteins which were identified in at least two biological replicates. Normalized spectral abundance factor values were chosen as a quantitative measure (Zybailov et al., 2006) for comparison. Proteins verified in S1 + S2 search and categorized as nuclear (and accordingly considered chromosomal) represented 30% of all repetitive IDs for the flow-sorted chromosomes. This was significantly more than ~10% obtained for the control (i.e., the original root-tip homogenate) and the chromosome-depleted fraction. Data analysis confirmed the expected enrichment of nuclear/chromosomal proteins in chromosomes as the percentages for individual search categories were rather similar for all three sample types when comparing the numbers of protein IDs (Figure 4). Non-nuclear proteins always represented more than 80% of IDs, and almost 90% were identified in the chromosome-depleted fraction. The category NUCLEAR S2 was the most enriched one and contained histones categorized according to Arabidopsis homology as histones and their variants: H2 (13 IDs), H1 (six IDs), and H3 (three IDs). Next, four DNA helicases were found although three of them are classified as DNA replication licensing factor or minichromosome maintenance (MCM) proteins. Single SMC protein and DNA (cytosine-5)-methyltransferase CHROMOMETHYLASE 3 (CMT3; EC 2.1.1.37) were found in this category, which may reflect the under-representation of characterized barley representatives in the database. Additionally, three chromatin handling proteins, chromatin-remodeling ATPase (2 IDs) and facilitates chromatin transcription complex subunit SSRP1 protein, confirm the presence of predominantly well-characterized DNA-binding proteins or enzymes in this group.

Altogether, a combination of the strong cation exchange (SCX) and MG-related analyses provided a list of 837 unique IDs, which may be considered nuclear/chromosomal based on the applied bioinformatics processing (Supplementary Table S5). This group of identified proteins was compared with the content of the UNcleProt barley nuclear protein database (Blavet et al., 2017). Only 311 out of the 837 proteins had matches in the database. Table 2 shows that a majority of them, categorized by searches according to their names and functional annotations, were DNA-associated proteins (including histones) and RNA-associated proteins as well as proteins attributed to ribosomes. Numerous matched IDs were uncharacterized proteins in the barley proteome but could be assigned by homology to their Arabidopsis counterparts. Many novel protein IDs outside the UNcleProt belonged to the same categories but above that the others were typically chromosomal (e.g., condensin, cohesin, and kinetochore components) or mitosis-related (kinesins).

**Table 2:** Attributes assigned to the 837 identified barley chromosomal proteins (NUCLEAR S1 + S2).

Searched text string	Novel IDs in chromosomes		Matched nuclear IDs	
	HORVU^a^	ARATH^b^	HORVU^a^	ARATH^b^
Chromosome	11	20	3	13
Chromatin	2	22	2	8
DNA	17	41	17	35
Kinetochor	0	3	0	1
Histon	52	61	62	76
Replicat	6	11	2	11
Mitotic	0	1	0	2
Kinesin	10	12	0	0
Condensin	5	4	0	0
Cohesin	0	4	0	2
Transcript	0	18	3	13
RNA	11	54	7	36
Ribosome	16	35	19	34
Uncharacterized	185	1	88	2

The text strings provided in the first column were applied as “keywords” for searching in the names of barley or homologous Arabidopsis proteins (see Supplementary Table S5). Only 311 out of the 837 proteins matched the original dataset of the barley nuclear protein database UNcleProt (Blavet et al., 2017). The others were thus considered novel IDs.

a HORVU, *Hordeum vulgare*.

b ARATH, *Arabidopsis thaliana*.

### Localization of FIB1 on Mitotic Chromosomes

**Figure 5:**
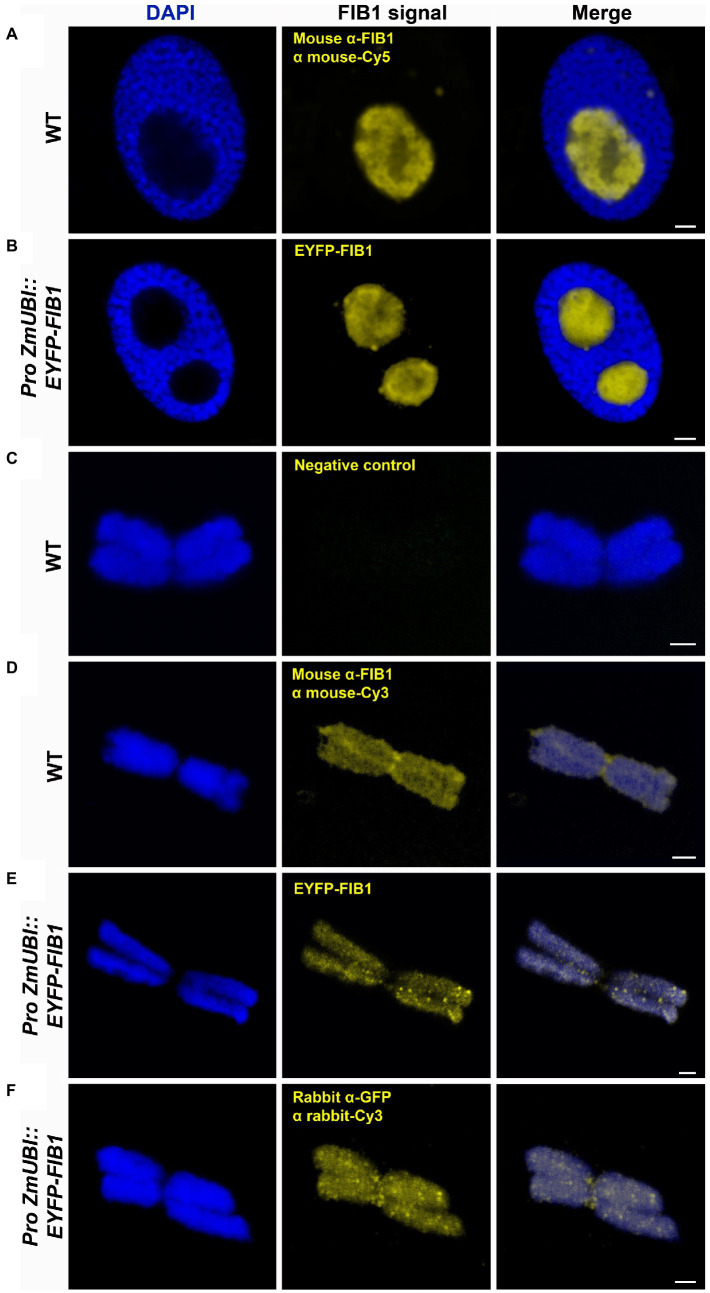
Detection of barley FIB1 in interphase nuclei and on metaphase chromosomes. All nuclei and chromosomes were counterstained with DAPI. Unstained regions within interphase nuclei correspond to nucleoli. **(A)** Wild-type (WT) interphase nucleus with FIB1 detected *via* immunolocalization with a specific antibody against FIB1 and secondary fluorochrome-coupled antibody. **(B)** The interphase nucleus of the barley reporter line expressing a translational fusion of the EYFP-FIB1. **(C)** Metaphase chromosome without immunostaining serving as a negative control for autofluorescence in Cy3 channel. **(D)** WT metaphase chromosome with FIB1 detected as described in **(A)**. **(E)** Reporter line metaphase chromosome with direct EYPF-FIB1 signal. **(F)** Reporter line chromosome with EYFP-FIB1 signal enhanced *via* immunolocalization with anti-GFP-Cy3 antibody (recognizing also EYFP). Scale bars = 2 μm.

Besides the known chromatin proteins, the SCX and MG identified a high number of chromosomal proteins that are not associated with chromatin. A prominent group was represented by nucleolar proteins, including abundant peptides from FIB1. FIB1 is a marker of nucleoli that forms foci of various densities. We have confirmed the localization of FIB1 in nucleoli of barley interphase nuclei by immunostaining and also by constructing a barley reporter line constitutively expressing a translational fusion of EYFP-FIB1 (Figures 5A,B). To confirm FIB1 localization on mitotic chromosomes as suggested by the proteomic analysis, we flow-sorted metaphase chromosomes of wild-type and EYFP-FIB1 reporter line into microscopic slides and observed them either directly (EYFP-FIB1) or after immunodetection with the antibodies against FIB1 and/or GFP (recognizes also EYFP). In all cases, a signal was observed confirming the presence of FIB1 (native or fusion) protein, which was not the case for negative controls when chromosomes were incubated only with a secondary antibody (Figures 5C–F). The chromosomes were covered entirely with foci of higher signal intensity. On some chromosomes, we observed even FIB1 localization in the kinetochore-binding region (Figure 5D). This observation confirmed that nucleolar protein FIB1 is associated with plant mitotic chromosomes during cell division.

**Figure 6:**
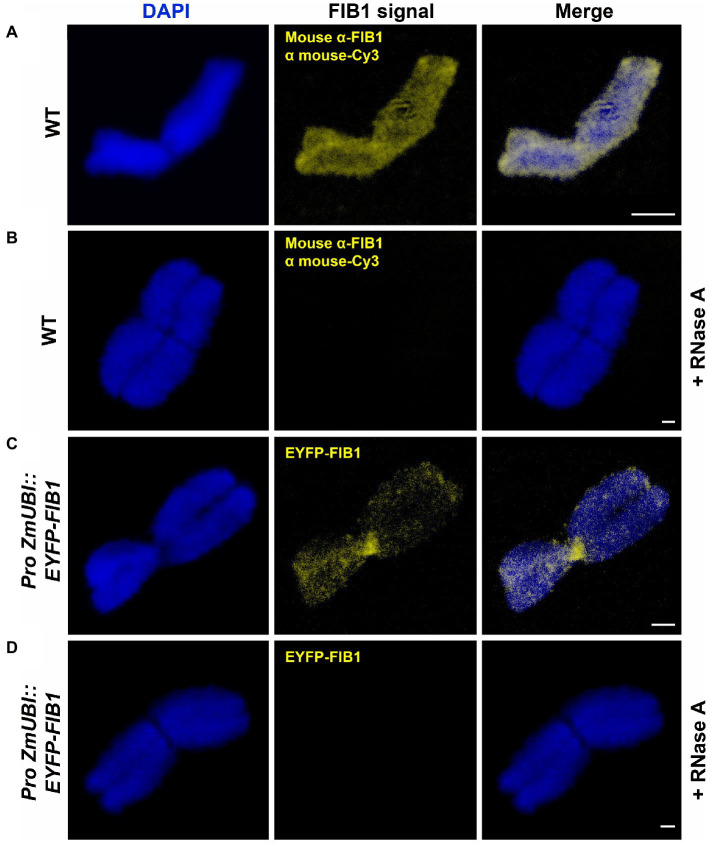
FIB1 is removed from chromosomes by RNase A treatment. All chromosomes were counterstained with DAPI. **(A)** WT flow-sorted chromosome with immunolocalized FIB1. **(B)** Representative chromosome prepared in the same way with additional RNAse A treatment. **(C)** Chromosome from a transgenic reporter line expressing EYFP-FIB1 fusion protein. **(D)** Chromosome from the same material as in **(C)** with additional RNAse A treatment. Scale bars = 2 μm.

FIB1 is an RNA methyltransferase that functions in complex with other proteins and RNA molecules. Therefore, we asked whether FIB1 is localized on chromosomes as an isolated protein or in complex with RNA. To test this, we treated flow-sorted chromosomes by RNase (Figure 6). In both cases, immunolocalized native FIB1 and EYFP-FIB1 fusion protein, RNase A treatment led to the loss of FIB1 signals, suggesting that the entire FIB1 complex including RNA molecules is associated with barley mitotic chromosomes.

## Discussion

### Flow Cytometry as a Critical Step in Plant Chromosomal Proteomics

We have identified the largest set to date of proteins associated with plant mitotic chromosomes. Barley was chosen as a model plant because its reference genome is available (Mascher et al., 2017) as well as a plethora of transcriptome data (Kintlová et al., 2017; Rapazote-Flores et al., 2019). Its nuclear proteome has been characterized as well (Petrovská et al., 2014; Blavet et al., 2017). Importantly, a well-established method is available for the preparation of suspensions of intact mitotic metaphase chromosomes and their purification by flow cytometric sorting (Lysák et al., 1999). This allowed us to prepare samples enriched for proteins from mitotic metaphase chromosomes. Vertebrate chromosomes, on the other hand, are commonly prepared by a density gradient centrifugation, for example, by applying sucrose and Percoll gradients (Samejima and Earnshaw, 2018). While highly synchronized mitotic cell populations have been used to characterize the proteome of human and animal chromosomes, such a synchrony is hardly reachable with plant tissues.

**Figure 7:**
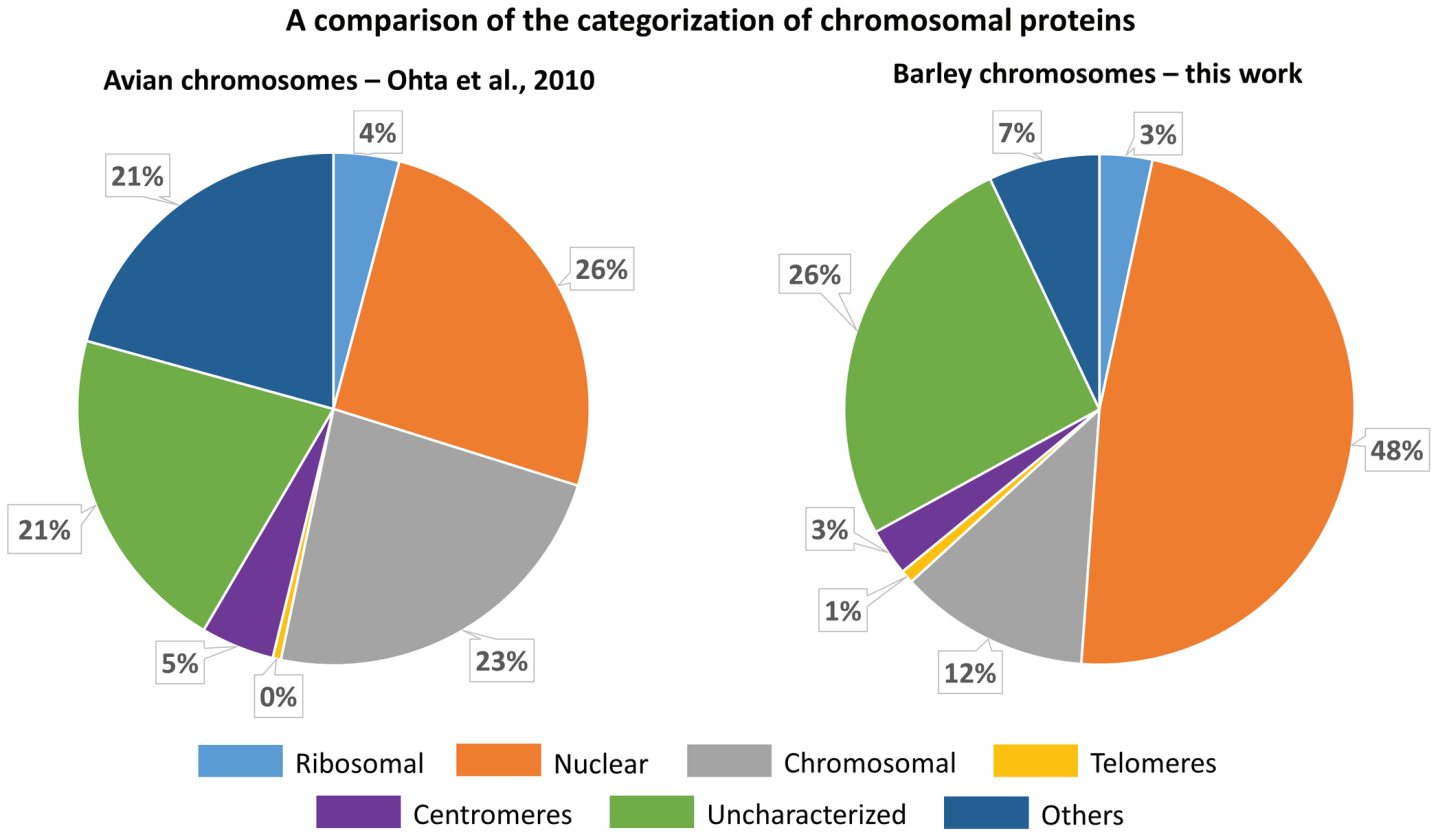
A comparison of the categorization of chromosomal proteins identified for chicken DT40 cells (Ohta et al., 2010) and flow-sorted barley chromosomes (this work). The categorization for barley is derived from information provided in the respective protein names and retrieved by searching in UniProt GO-CC, UniProt Subcellular Localization a DAVID GOTERM-CC data (see Supplementary Table S5).

As chromosomes are released into the cytoplasm during mitosis, it is critical to ensure that the chromosomal protein content is not contaminated by cytoplasmic proteins. As such a contamination cannot be *a priori* avoided, we have identified chromosomal proteins by comparing the results of protein identification in: (1) the original homogenate containing chromosomes plus cellular and tissue debris, (2) chromosomes purified by flow sorting, and (3) chromosome-depleted homogenate containing only cellular and tissue debris. Given that the protocol for preparation of chromosome suspensions (Lysák et al., 1999) includes mild formaldehyde fixation, there is a risk of crosslinking cytoplasmic proteins with those forming the perichromosomal layer. As this should be a random process, it should result in protein clusters of varying size irregularly associated with the chromosome surface. However, only highly regular structures were observed on the surface of flow-sorted barley chromosomes using environmental scanning electron microscopy (V. Neděla, personal communication). Based on this observation and our experimental design, we consider the results obtained in this work as well supported. We categorized all proteins identified in flow-sorted chromosomes using the information obtained from the relevant UniProtKB database records and related DAVID search data, and compared with a previous proteomics analysis of avian chromosomes (Ohta et al., 2010). The comparison showed a good overall agreement as the majority of proteins was classified as nuclear or chromosomal, while uncharacterized proteins represented consistently about 20–25% (Figure 7).

To assess barley chromosomal proteome from a biological point of view, we considered a semi-quantitative nature of our methods and looked at the most relevant proteins and complexes identified. These proteins were classified as nuclear/chromosomal and were ordered decreasingly according to the number of unique identified peptides and analyzed as regards to their biological role based on the existing annotation and homology to Arabidopsis.

### Pre-separations of Peptides Prior to nLC–MS/MS to Increase the Protein Identification Rate

In-gel digestion yielded only 63 proteins with 16 classified as nuclear/chromosomal. These proteins comprised almost exclusively histone proteins (H1 to H4) specific to both euchromatin (H3.3, H2A.XB, and H2A.Z) and heterochromatin (H3.1, H2A.W, and H1.2). The heterochromatic variants were generally more frequent, which may correspond to the high proportion of repetitive DNA in the barley genome (Baker et al., 2015). The GTP-binding protein RAN3 (Hv: M0UFI4; At: Q8H156/AT5G55190) was the only non-histone case likely responsible for nucleocytoplasmic protein transport. However, RAN3 most likely does not have a direct DNA-binding activity and the analysis in Arabidopsis identified it as interactor of METHYL-BINDING PROTEIN 5, which is one of four Arabidopsis MBDs binding to 5-methyl cytosine (Yano et al., 2006). In summary, the in-gel digestion method revealed practically only nucleosomal subunits, suggesting a loss of a majority of chromosomal proteins and/or a failure to detect them when using this approach.

The other two methods used, i.e., the SCX and C18 reversed-phase MG, were based on the in-solution isolated chromosomal proteins and differed in the principle of pre-separation of peptide mixtures. Consistently, around 15% of the obtained protein IDs were classified as nuclear/chromosomal. The lists of the most abundant proteins were very similar for both methods (Supplementary Tables 3 and 4). The four most common proteins/complexes (Group 1) were TOPOISOMERASE 2 (TOP2), POLY(ADP-RIBOSE) POLYMERASE 2 (PARP2), various histone proteins, and condensin complex subunits. At the fifth to the seventh position (Group 2), we found inner nuclear envelope protein CROWDED NUCLEI 1 (CRWN1), nucleolar proteins (e.g., FIB1), and subunits of the replication licensing complex MCM MCM2 to MCM7. The remaining positions (Group 3) were more variable between the methods and represented a mix of proteins with various chromatin-related functions. They included chromatin-remodeling ISWI complex factor (CHR11); FACT complex factors (SPT16 and SSRP1 subunits); high mobility group proteins; histone chaperone NAP1,2; DNA repair proteins ZINC 4 FINGER DNA 3’-PHOSPHOESTERASE (ZDP), LIGASE 1 (LIG1) and KU80; and transcriptional gene silencing factors CHG DNA methyltransferase CMT3, CG DNA METHYLTRANSFERASE 1 (MET1), or ARGONAUTE 4.

Based on the spectra of the most abundant chromosomal proteins, we can draw a picture of barley metaphase mitotic chromosome proteins. Using all three methods, we obtained abundant histone proteins, which are the expected component of the highly compact metaphase chromosomes. The frequent presence of histone H1.2 agrees with the transcriptionally inactive chromatin of condensed chromosomes. From the condensin complex, we found mainly the core subunits STRUCTURAL MAINTENANCE OF CHROMOSOMES 2 and 4 (SMC2 and SMC4) and there was only one hit for the cohesin complex, suggesting that the latter is less abundant. To our surprise, the most abundant peptides in both SXC and MG methods originated from TOP2. Although the TOP1 was present, it was less abundant. This indicates frequent sister chromatid intertwinings and/or supercoils that need to be mitigated primarily by the TOP2 and to a lesser extent by the TOP1 activities. The candidates from the Group 2 are intriguing as they represent typical interphase nuclear proteins. CRWN1 is an inner nuclear envelope (NE) protein that interacts with other chromatin-binding proteins and thus mediates chromatin and chromosome organization (Meier et al., 2016; Mikulski et al., 2019). It is tempting to speculate that the complex remains bound to the surface of the chromosome also during mitosis, helping to anchor the centromeric region to the NE. This could, on the one hand, accelerate the kinetics of the division and, on the other hand, help maintaining Rabl chromosome organization found in barley nuclei (Tiang et al., 2012).

A surprising observation concerned the numerous peptides derived from the maintenance complex 2 to 7 (MCM2-7). This complex is typical for DNA replication initiation and elongation during the S-phase of the cell cycle (Tuteja et al., 2011). Currently, no data support a direct role of the MCM2-7 complex during mitosis. Therefore, the MCM2-7 proteins may represent a contamination from the cytoplasm. However, the presence of some other (Group 3) proteins, such as DNA replication coupled maintenance DNA methyltransferases MET1 and CMT3, indicates that some replication-related processes appear during mitosis, possibly at specific DNA repair sites. Furthermore, there is a specific report of MCM function in late mitosis. Other members of Group 3 indicate active transcription (FACT and ISWI complex subunits) and DNA repair. From the DNA repair enzymes, we detected KU80, which acts as a heterodimer with KU70 and stabilizes free DNA ends. In addition, we found ZDP and LIG1, both acting in the excision repair pathways. This indicates a repair of DNA double and single-strand breaks that could arise from the tension during chromosome condensation and/or topoisomerase activity.

### Validation of Perichromosomal Location of FIB1

Abundant nucleolar proteins bind to chromosomes after nucleoli disassemble at the onset of mitosis. Several studies have demonstrated the presence of nucleolar proteins over the entire mitosis and their important role in reconstituting a new nucleolus after the mitosis is completed (reviewed in Kalinina et al., 2018). Our proteomic data confirm the idea that at least part of these nucleolar proteins is physically attached to plant mitotic chromosomes, where they presumably contribute to the formation of a perichromosomal layer. We have experimentally validated this localization for the large nucleolar protein FIB1 using multiple approaches. FIB1 is a part of small nuclear ribonucleoprotein complexes involved in the first steps of RNA splicing and processing pre-ribosomal (r)RNAs (Reichow et al., 2007). Sirri et al. (2016) demonstrated that precursor rRNAs associate with the perichromosomal layer of human chromosomes where they serve as binding sites for various nucleolar proteins. In our work, the treatment of barley chromosomes with RNase A resulted in a strong reduction of FIB1 signal. This observation supports the critical role of RNAs in the assembly of perichromosomal layer also in plants and confirms the specific binding of FIB1. The marker of proliferation Ki67 is another nucleolar protein associating with perichromosomal layer in human (Takagi et al., 1999). According to Hayashi et al. (2017), Ki67 functions as a binding scaffold for pre-RNAs to which nucleolar proteins bind. Given the critical role of Ki67 in human, it is surprising that our analyses did not identify Ki67 in the proteome of barley chromosomes. Given the large evolutionary distance between animals and plants, it is possible that a similar role is played by a different and not yet described protein.

## Conclusion

Our results provide valuable insights into the protein composition of condensed barley chromosomes and support a multi-layer model suggested for human mitotic chromosome (Uchiyama et al., 2005; Takata et al., 2007). This model categorized the identified proteins into separate groups: (1) coating cytoplasmic proteins on chromosome surfaces, (2) a perichromosomal layer comprising RNAs and nucleolar proteins, and (3) chromosome structural and fibrous proteins deeper in the chromosome core. Indeed, we detected the presence of many cytoplasmic proteins in the sorted mitotic barley chromosomes. However, these were excluded by our multi-classifier data analysis as random cellular hitchhikers with no essential functions during mitosis. On the other hand, a large group of nucleolar proteins was assigned as truly chromosomal and this finding, together with an important organizational role of RNA, was further confirmed by immunolocalization experiments. Finally, we included into the list a variety of proteins contributing to the processes of chromosome organization and maintenance. Generally, there were attempts to assign the identified barley proteins to their counterparts in Arabidopsis. In some cases, we could find a high homology for relevant hits supported by experimental data in the literature. Examples are SWITCH/SUCROSE NONFERMENTING (SWI/SNF) chromatin-remodeling complex proteins. Barley SNF protein, UniProtKB access. no. A0A287IBE5, shows 75% sequence similarity to its ARATH homolog Q9FMT4. Barley SWI3C subunit (access. no. A0A287QVR1) is identical at 46%. The possible regulatory function of Arabidopsis SWI3C resides in affecting plant development as its mutations led to lower fertility (Sarnowski et al., 2005). Barley PROLIFERATING CELL NUCLEAR ANTIGEN 2 (PCNA2), access. no. A0A287FZQ3, is largely homologous (sequence similarities above 80%) to Arabidopsis (Q9ZW35) and human PCNAs (Q6FHF5). This protein is an auxiliary component for DNA polymerase delta and is involved in the replication control. Its interaction partner REPLICATION FACTOR C PROTEIN SUBUNIT 1, which participates in meiotic recombination and crossover formation process (Liu et al., 2013), was identified in several forms in the present proteomics dataset. As exemplified by the missing counterpart of human Ki67, many chromosome-associated proteins that play key roles in plant mitotic pathways remain elusive. Thus, our dataset may serve as a valuable resource for functional characterization of plant chromosomal proteins, their comparative phylogenetic analyses, and ultimately, the development of the next-generation models for the hierarchical organization of plant chromosomes.

## Data Availability Statement

The datasets presented in this study can be found in the PRIDE Archive (https://www.ebi.ac.uk/pride/archive/; Perez-Riverol et al., 2019). The accession number is PXD024689.

## Author Contributions

BP and JV maintained barley plants and purified mitotic chromosomes by flow cytometric sorting. ZP performed all experiments comprising microgradient pre-separation of peptides, he also processed, analyzed, and curated the data, created all figures, and contributed to the original manuscript draft writing. JB carried out all experiments that included in-gel digestion and in-solution digestion followed by SCX pre-separation of peptides. IC partly designed the study and employed the methodologies, he also created the tables and contributed to data processing and writing of the original manuscript draft. RL was responsible for all MS analyses. KK prepared barley transgenic line and performed immunostaining and microscopy. VB coordinated barley transformation. AP evaluated and discussed the biological meaning of the obtained results. JD, MŠ, and AP conceived, conducted, and supervised the study and secured funding. MŠ wrote the original manuscript draft. All authors read, edited, and approved the final manuscript.

## Conflict of Interest

The authors declare that the research was conducted in the absence of any commercial or financial relationships that could be construed as a potential conflict of interest.

## Publisher’s Note

All claims expressed in this article are solely those of the authors and do not necessarily represent those of their affiliated organizations, or those of the publisher, the editors and the reviewers. Any product that may be evaluated in this article, or claim that may be made by its manufacturer, is not guaranteed or endorsed by the publisher.

## Abbreviations

ACN: acetonitrile
ARATH: *Arabidopsis thaliana*
CCAN: centromere-associated network
DAPI: 4',6-diamidine-2'-phenylindole
DTT: dithiothreitol
ESI: electrospray ionization
EYFP: enhanced yellow fluorescent protein
FDR: false discovery rate
FoA: formic acid
GFP: green fluorescent protein
GO: gene ontology
HMG: high mobility group
HORVU: *Hordeum vulgare*
HyD: hybrid detectors
ID: identification
KMN: Knl1, Mis12, and Ndc80 network
nLC: nanoflow liquid chromatography
MALDI: matrix-assisted laser desorption/ionization
MCM: minichromosome maintenance
MG: microgradient
MGF: Mascot generic format
MS: mass spectrometry
MS/MS: tandem mass spectrometry
NSAF: normalized spectral abundance factor
NWC: NOL11, WDR43, and Cirhin complex
PMSF: phenylmethylsulfonyl fluoride
RAF-BT: raffinose-modified bovine trypsin
SDS-PAGE: sodium dodecyl sulfate–polyacrylamide gel electrophoresis
SCX: strong cation exchange
SMC: Structural maintenance of chromosome
TCEP: tris(2-carboxyethyl)phosphine
TFA: trifluoroacetic acid
WT: wild type
